# Amblyopia risk factors in congenital nasolacrimal duct obstruction: A longitudinal case-control study

**DOI:** 10.1371/journal.pone.0217802

**Published:** 2019-06-13

**Authors:** YungJu Yoo, Hee Kyung Yang, Namju Kim, Ho-Kyoung Choung, Jeong-Min Hwang, Sang-In Khwarg

**Affiliations:** 1 Department of Ophthalmology, Kangwon National University School of Medicine, Chuncheon, Korea; 2 Department of Ophthalmology, Seoul National University College of Medicine, Seoul, Korea; 3 Department of Ophthalmology, Seoul National University Bundang Hospital, Seongnam, Korea; 4 Department of Ophthalmology, Seoul Municipal Government-Seoul National University Boramae Medical Center, Seoul, Korea; 5 Department of Ophthalmology, Seoul National University Hospital, Seoul, Korea; Faculty of Medicine, Cairo University, EGYPT

## Abstract

**Purpose:**

To investigate longitudinal changes in risk factors for amblyopia in children treated with congenital nasolacrimal duct obstruction (CNLDO).

**Methods:**

Retrospective observational case control study. A total of 446 children under 4 years of age who underwent probing and/or intubation for CNLDO between January 2004 and January 2018, and 446 age-matched controls were included. Cycloplegic refraction and ocular alignment were investigated at the time of treatment and after at least one year of symptom improvement. Children were classified as having amblyopia risk factors on the basis of the American Association for Pediatric Ophthalmology and Strabismus guideline in 2013.

**Main outcome measures:**

The prevalence of amblyogenic refractive errors, and determinants associated with the presence of amblyogenic refractive errors in CNLDO patients.

**Results:**

The prevalence of amblyogenic refractive errors in CNLDO patients (5.4%) was similar to that of the control group (6.5%) (P = 0.571). After one year of symptom improvement in CNLDO patients, the prevalence of amblyogenic refractive errors was 4.7%. There was no difference in the prevalence of amblyogenic refractive errors between unilateral and bilateral CNLDO patients. Multivariate analysis revealed that manifest strabismus was the only risk factor related with the presence of amblyogenic refractive errors (odds ratio = 6.383, confidence interval = 1.205–33.826, P = 0.029).

**Conclusions:**

This study found no evidence to suggest that the prevalence of amblyopia risk factors is higher in CNLDO patients compared with normal controls. Manifest strabismus was the only determinant associated with the presence of amblyogenic refractive errors.

## Introduction

Congenital nasolacrimal duct obstruction (CNLDO) appears in 5 to 15% of full-term infants [1, 2]. CNLDO is characterized by constant tearing and intermittent discharge occurring in one or both eyes [1, 2]. Several authors have reported a possible association between CNLDO and amblyogenic refractive errors, and it is generally accepted that the risk for amblyopia is higher in patients with CNLDO [3–6]. Persistent tearing induces blurred vision that interferes with emmetropization resulting in anisometropia which is a strong amblyogenic risk factor [3].

Among previous studies conducted on CNLDO patients, no large-scale study has evaluated the prevalence of risk factors for amblyopia in patients with CNLDO who underwent probing and/or intubation [3, 4, 7–11]. A better understanding of the effects of CNLDO on amblyopia can be obtained by longitudinal observations of amblyopia risk factors, especially after symptom improvement. Furthermore, most of the previous reports did not include a control group [7, 9–13]. Only one study compared the prevalence of amblyopia between CNLDO and controls [6], however, CNLDO spontaneously resolved without treatment in most patients which leaves some doubt regarding the effect of CNLDO on the development of amblyopia.

Therefore, we aimed to investigate longitudinal changes in the prevalence of risk factors for amblyopia among patients with CNLDO after treatment, and to evaluate the clinical characteristics associated with amblyogenic refractive errors in this patient population.

## Materials and methods

The institutional review board of Seoul National University Bundang Hospital approved the study (B-1802-453-102). This study was carried out in accordance with the recommendations of the Declaration of Helsinki for biomedical research involving human subjects. Informed consent was not given, as all patient records were provided to/accessed by the authors in a fully anonymized format. The institutional review board of Seoul National University Bundang Hospital waived the requirement for informed consent of parents.

### Patients

We performed a retrospective review of patients with CNLDO under 4 years of age, who underwent either probing or intubation (by NK or HKJ) at Seoul National University Bundang Hospital, and Seoul Municipal Government-Seoul National University Boramae Medical Center between January 2004 and January 2018. A routine ophthalmologic examination including cycloplegic refraction and ocular deviation measurement was performed at the time of treatment and during annual follow-up examinations afterwards. Exclusion criteria were as follows; prematurity (< 37 weeks gestational age), low birth weight (1,500 to 2,499 g) [14], and any ocular deformity such as ptosis or keratopathy which could affect refractive errors. Children with systemic syndromes, severe mental disability [15], craniosynostosis [16], or hydrocephalus [16], which are known to be related to refractive errors were also excluded [17].

We randomly selected an age-matched control group from patients who visited the outpatient clinic of Seoul National University Bundang Hospital for routine eye examination. Children without CNLDO and with no ocular disease other than refractive errors were included. When both eyes of a patient met the eligibility criteria of age-matched controls, one eye was randomly selected for statistical analysis.

### Clinical evaluation

Data were collected on demographics and clinical characteristics, including patient’s gestational age, birth weight, gender, age at diagnosis, number of probing and/or intubation, and age of treatment. Medical records were reviewed for ocular alignment and motility, cycloplegic refractive errors, and anterior and posterior segment findings. The initial refractive errors were recorded during the first ocular examination for all patients with cycloplegic refraction. Cycloplegic refraction was performed by waiting for 30–40 minutes after applying 1% cyclopentolate three times in each eye every 5 minutes. Ocular deviation was measured with the alternate prism and cover test (APCT), or the Krimsky test for subjects unable to perform the APCT. The final refractive errors were defined as the most recent cycloplegic refraction performed at least one year after symptom relief. We investigated the prevalence of risk factors for amblyopia based on the American Association for Pediatric Ophthalmology and Strabismus (AAPOS) guideline, revised in 2013 (Table 1) [18].

**Table 1:** Amblyopia risk factors based on the American Association for Pediatric Ophthalmology and Strabismus (AAPOS) original referral criteria in 2013.

	Refractive amblyopia risk factor targets*			
Age, months	Astigmatism	Hyperopia	Anisometropia	Myopia
12–30	> 2.0D	> 4.5D	> 2.5D	> -3.5D
31–48	> 2.0D	> 4.0D	> 2.0D	> -3.0D
	Non-refractive amblyopia risk factor targets^†^			
All ages	Manifest strabismus > 8 PD in primary position Media opacity > 1 mm			

D = diopters; PD = prism diopters.

* Additional reporting of sensitivity to detect greater-magnitude refractive errors is encouraged.

† For all ages.

### Data analysis and statistics

For statistical analysis, SPSS ver. 21.0 software (IBM Corporation, Armonk, NY, USA) was used. Demographic data (e.g., gender and age) were compared between CNLDO patients and controls using the chi-square test and the unpaired t test. Comparison between patients with CNLDO versus (vs.) controls and unilateral vs. bilateral CNLDO, were assessed using the independent t test or Pearson correlation test for continuous variables and chi-square test for categorical variables, as applicable. Bivariate logistic regression was performed in terms of the presence of amblyogenic refractive errors as the dependent variable to evaluate the relationship between related variables. Predictors with a p-value of 0.1 or less in univariate analysis were included as a candidate in multivariate analysis. A p-value < 0.05 was considered statistically significant. Data are presented as mean ± standard deviation unless stated otherwise.

## Result

### Subject characteristics

This study initially enrolled 1037 patients with CNLDO who underwent treatment; 591 were excluded due to incomplete ocular examination (n = 526), out-of-age criteria (n = 27), systemic disease or severe mental disability (n = 19), uncertain diagnosis (n = 12), accompanying ocular abnormalities (n = 4), and prematurity (n = 3). The remaining 446 (43.5%) patients with CNLDO and 446 age-matched controls were evaluated; their demographic information and clinical characteristics are summarized in Table 2. The mean age of CNLDO patients and controls at the initial examination were not significantly different (1.8 ± 0.9 vs. 1.7 ± 1.1 years, respectively; P = 0.792). Among 446 CNLDO patients, 96 patients (21.5%) had bilateral CNLDO. For treatment options, 431 patients (96.6%) underwent probing as the first treatment and 38 (8.5%) underwent intubation. The average number of probing was 1.6 ± 1.2 (range, 1–9), and 167 (37.4%) patients underwent probing more than once.

**Table 2:** Clinical characteristics and treatment of 446 children diagnosed with congenital nasolacrimal duct obstruction.

Findings	Value
Gender N, (%)	
Male	243 (54.5)
Female	203 (45.5)
Age at first examination (years)	1.8 ± 0.9
Age at first probing (years) (n = 431)	1.2 ± 0.6
Age at first surgery (years) (n = 38)	2.3 ± 1.3
Laterality of CNLDO diagnosis N, (%)	
Unilateral	350 (78.5)
Right	177 (39.7)
Left	173 (38.8)
Bilateral	96 (21.5)
CNLDO treatment N, (%)	
Probing only	408 (91.5)
Probing and intubation	23 (5.2)
Intubation only	15 (3.4)
Number of probing procedures N, (%)	1.6 ± 1.2
1 procedure	264 (59.2)
2 procedures	100 (22.4)
≧ 3 procedures	67 (15)

CNLDO = congenital nasolacrimal duct obstruction; N = numbers

### Comparison of CNLDO patients and age-matched controls

Overall, CNLDO patients had a similar prevalence of amblyogenic refractive errors compared with controls (5.4% vs. 6.5%, respectively; P = 0.571) (Table 3). There was also no significant difference in the prevalence of each type of refractive errors between CNLDO patients and controls (all P>0.5) (Table 3). With regard to strabismus, 10 (2.2%) patients exhibited manifest strabismus over 8 prism diopters in the primary position. Among all patients, a follow-up examination including cycloplegic refraction was performed in 148 patients (33.2%) after at least one year of symptom resolution. The mean age at final examination was 3.2 ± 0.6 years, and seven (4.7%) patients exhibited amblyogenic refractive errors. There was no significant change in the prevalence of amblyogenic refractive errors at the final follow-up examination (n = 7 [4.7%]) compared with the initial examination (n = 19 [4.3%]).

**Table 3:** Comparison of the prevalence of amblyogenic refractive errors between congenital nasolacrimal duct obstruction patients and age-matched controls.

	Patients (N = 446)	Controls (N = 446)	P value*
Amblyogenic refractive errors N, (%)	24 (5.4)	29 (6.5)	0.571
Hyperopia	5 (1.1)	8 (1.8)	0.578
Myopia	4 (0.9)	2 (0.4)	0.686
Astigmatism	15 (3.4)	16 (3.6)	0.855
Anisometropia	4 (0.9)	4 (0.9)	0.999

N = numbers

* P value by chi-square test

### Comparison of unilateral and bilateral CNLDO

Among 446 patients with CNLDO, 96 (21.5%) had bilateral CNLDO. The mean age of treatment was not significantly different between patients with unilateral CNLDO and bilateral CNLDO (1.3 ± 0.8 vs. 1.2 ± 0.6 years, respectively; P = 0.119). According to treatment, there was no difference in the number of probing procedures between the two groups (1.6 ± 1.2 vs. 1.7 ± 1.0, respectively; P = 0.256). Following the 2013 AAPOS guidelines [18], 16 (4.6%) patients with unilateral CNLDO and eight (8.2%) patients with bilateral CNLDO exhibited amblyogenic refractive errors (P = 0.303). There was no difference in the rate of anisometropia between patients with unilateral (n = 2 [0.6%]) and bilateral (n = 2 [2.1%]) CNLDO (P = 0.170).

The interocular differences of spherical equivalent (SE) refractive errors and astigmatism were compared in 350 children with unilateral CNLDO. There was no significant difference in SE refractive errors (D, diopters) (P = 0.793) and astigmatism between the affected eye (2.00 ± 1.09 D) and unaffected eye (1.45 ± 1.03 D; P = 0.156) (Table 4). In patients with unilateral CNLDO, 6 of 16 cases (37.5%) with amblyogenic refractive errors exhibited greater hyperopia in the affected eye compared with the fellow eye (Table 4).

**Table 4:** Refractive errors and anisometropia in 350 patients with unilateral CNLDO.

	Affected eye		Unaffected eye		P value
SE refractive errors (D)	0.89 ± 4.02 (-7.88, 10.00)		0.52 ± 3.82 (-8.50, 9.00)		0.793*
Astigmatism (D)	2.00 ± 1.09 (0, 3.50)		1.45 ± 1.03 (0, 3.25)		0.156*

CNLDO = congenital nasolacrimal duct obstruction; SD = standard deviation; SE = spherical equivalent; D = diopters

* P value by paired t test

### Factors associated with risk factors for amblyopia

Table 5 summarizes factors associated with the presence of amblyogenic refractive errors in patients with CNLDO. In univariate analysis, performing intubation (odds ratio [OR] 4.264, 95% confidence interval [CI] 1.446–12.570; P = 0.009) and the presence of manifest strabismus (OR 6.162, 95% CI 1.215–31.248; P = 0.028) were significantly associated with the presence of amblyogenic refractive errors. In multivariate logistic regression models, in which no significant effects were eliminated, only the presence of manifest strabismus was significantly associated with the presence of amblyogenic refractive errors (OR 6.383, 95% CI 1.205–33.826; P = 0.029) (Table 5).

**Table 5:** Predictors of amblyogenic refractive errors in congenital nasolacrimal duct obstruction.

	Univariate analysis			Multivariate analysis		
	Odds Ratio	95% CI	P value	Odds Ratio	95% CI	P value
Age at diagnosis, *per 1 year later*	1.527	0.970, 2.402	0.067	1.184	0.689. 2.035	0.540
Age at examination, *per 1 year later*	1.227	0.745, 2.021	0.422			
Gender (male)	1.856	0.692, 4.974	0.219			
Unilateral CNLDO	2.184	0.836, 5.709	0.111			
Number of probing	1.064	0.748, 1.513	0.731			
Performing silicone tube intubation	**4.264**	**1.446, 12.570**	**0.009**	3.507	0.947, 12.987	0.060
Manifest strabismus	**6.162**	**1.215, 31.248**	**0.028**	**6.383**	**1.205, 33.826**	**0.029**

CI = confidence interval; CNLDO = congenital nasolacrimal duct obstruction

Factors with statistical significance are shown in boldface.

## Discussion

The prevalence of amblyogenic refractive errors in the general population ranges from 10.9% to 20.2% [19–22]. The present study demonstrated that 5.4% of children with CNLDO exhibited amblyogenic refractive errors, which was similar to the frequency (6.5%) in normal age-matched controls. Furthermore, this was the first study to determine longitudinal changes in the prevalence of amblyogenic refractive errors in CNLDO patients who were successfully treated. The prevalence of amblyogenic risk factors did not change after at least one year of symptom relief. Finally, the presence of manifest strabismus was the only factor associated with amblyogenic refractive errors in children with CNLDO.

Previous studies have been inconclusive about the relationship between CNLDO and amblyopia. Some reported an increased risk for amblyopia in CNLDO patients [3–5, 9, 12], while others did not [6, 11]. It has been suggested that persistent tearing and discharge leads to blurring of vision and interferes with clear focusing of images on the retina, which is critical for emmetropization [5]. In contrast, in a case-control cohort study, Ellis et al.[6] found no evidence to prove that persistent tear film interferes with emmetropization or the development of ocular alignment in CNLDO patients. A previous study examined visual acuity and refractive errors in CNLDO patients after more than 4 years of symptomatic improvement [6]. However, there was no detailed description of the severity of CNLDO and surgical treatment, as well as the prevalence of amblyogenic risk factors according to specific guidelines [6]. To overcome these limitations, in the present study, we limited subjects to children with persistent tearing that required probing or intubation.

Matta et al.[4] reported that 22% of children under 3 years of age with CNLDO exhibited risk factors for amblyopia as defined by the AAPOS vision screening guidelines revised in 2003 [23]. However, the guideline proposed by the AAPOS vision screening committee had not considered age-dependent changes of risk factors for amblyopia [18, 23]. That is why older guidelines have a high over-referral rate and significant discrepancy between the rate of amblyopia and that of amblyopia risk factors [13, 24, 25]. Therefore, we used the newly adjusted guidelines incorporating age-related changes of amblyopia risk factors [18].

Unilateral CNLDO showed significant association with anisometropia compared with bilateral CNLDO in previous reports [5, 7, 10]. Clinically significant anisometropia is well documented as a critical cause of amblyopia [26]. A previous report revealed that anisometropic amblyopia developed in 5 out of 130 patients with unilateral CNLDO and all had amblyopia on the same side of CNLDO [27]. The study suggested that image deformation caused by mucopurulent discharge and excessive tear film in the early stage of vision development could cause a lack of proper emmetropization [7, 27]. However, in the current study, the prevalence of anisometropia in both unilateral and bilateral CNLDO were similar to what is expected in the general population of children, which is contrary to previous reports [5, 7, 10]. This discrepancy is mainly due to different definitions of amblyopia risk factors among studies. Previous studies defined amblyopia risk factors based on the AAPOS guideline in 2003 [3–5, 9, 12], while our definition was based on the revised guideline in 2013 [18].

In the present study, manifest strabismus was the only significant factor associated with the presence of amblyogenic refractive errors. This is in line with previous reports that showed an association between esotropia and hyperopia/anisometropia [28–30]. Our finding supports the hypothesis that anisometropia and hyperopia, as well as pronounced astigmatism, may trigger the development of strabismus in susceptible patients owing to disturbances of fusion [30, 31].

This study has a few limitations. Firstly, selection bias can occur in a retrospective study. Follow-up was determined at the discretion of the examiner, and compliance was dependent on the patient’s caregiver. As a result, only 30.9% of the original CNLDO population completed follow-up examinations. Among the 19 patients with amblyogenic risk factors at the initial examination, only four patients performed the follow-up examination. This may have caused underestimation of the actual prevalence of amblyopia in this cohort. However, in order to reduce possible errors, comparison of prevalences between CNLDO patients and controls were determined if amblyogenic risk factors were present at least once at the initial and/or final examinations. Nevertheless, the prevalence of amblyogenic risk factors revealed no statistical difference between patients and controls. Second, the control group was not recruited from the general population, but from those who were referred to our hospital for routine eye examination and had no amblyogenic risk factors including manifest strabismus. Thus, we do not know whether the prevalence of strabismus is higher in patients with CNLDO compared to those without CNLDO. However, the literature addressing the prevalence of childhood strabismus is substantial, [32–34] and our study population demonstrated similar values compared with previous studies. In addition, the risk factors for amblyopia in CNLDO patients are mainly related to refraction. Therefore, this does not change our qualitative conclusions. Finally, because all study patients were Koreans, extrapolating these results to other populations may be problematic.

In conclusion, in the present study, we demonstrated that there is no difference in the prevalence of amblyogenic refractive errors in patients with CNLDO compared with age-matched controls, and this tendency did not change even after one year of symptom resolution.

## References

[1] Lee SY, Chung HS, Kim HB, Namgung R, Han DG. The incidence of congenital nasolacrimal duct obstruction in Korean neonates. J Korean Ophthalmol Soc. 1989;30(1):5–8.

[2] MacEwen CJ, Young JD. Epiphora during the first year of life. Eye (Lond). 1991;5 (Pt 5):596–600. 10.1038/eye.1991.103 1794426

[3] Piotrowski JT, Diehl NN, Mohney BG. Neonatal dacryostenosis as a risk factor for anisometropia. Arch Ophthalmol. 2010;128(9):1166–1169. 10.1001/archophthalmol.2010.184 20837801

[4] Matta NS, Singman EL, Silbert DI. Prevalence of amblyopia risk factors in congenital nasolacrimal duct obstruction. J AAPOS. 2010;14(5):386–388. 10.1016/j.jaapos.2010.06.012 21035062

[5] Matta NS, Silbert DI. High prevalence of amblyopia risk factors in preverbal children with nasolacrimal duct obstruction. J AAPOS. 2011;15(4):350–352. 10.1016/j.jaapos.2011.05.007 21907117

[6] Ellis JD, MacEwen CJ, Young JD. Can congenital nasolacrimal-duct obstruction interfere with visual development? A cohort case control study. J Pediatr Ophthalmol Strabismus. 1998;35(2):81–85. 9559506 10.3928/0191-3913-19980301-05

[7] Kipp MA, Kipp MA Jr., Struthers W. Anisometropia and amblyopia in nasolacrimal duct obstruction. J aapos. 2013;17(3):235–238. 10.1016/j.jaapos.2012.11.022 23602456

[8] Saleem AA, Siddiqui SN, Wakeel U, Asif M. Anisometropia and refractive status in children with unilateral congenital nasolacrimal duct obstruction. Taiwan J Ophthalmol. 2018;8(1):31–35. 10.4103/tjo.tjo_77_17 29675347 PMC5890582

[9] Simon JW, Ngo Y, Ahn E, Khachikian S. Anisometropic amblyopia and nasolacrimal duct obstruction. J Pediatr Ophthalmol Strabismus. 2009;46(3):182–183. 19496504 10.3928/01913913-20090505-14

[10] Siddiqui SN, Mansoor H, Asif M, Wakeel U, Saleem AA. Comparison of Anisometropia and Refractive Status in Children With Unilateral and Bilateral Congenital Nasolacrimal Duct Obstruction. J Pediatr Ophthalmol Strabismus. 2016;53(3):168–172. 10.3928/01913913-20160405-06 27224951

[11] Ramkumar VA, Agarkar S, Mukherjee B. Nasolacrimal duct obstruction: Does it really increase the risk of amblyopia in children? Indian journal of ophthalmology. 2016;64(7):496–499. 10.4103/0301-4738.190101 27609160 PMC5026073

[12] Kim JW, Lee H, Chang M, Park M, Lee TS, Baek S. Amblyopia risk factors in infants with congenital nasolacrimal duct obstruction. J Craniofac Surg. 2013;24(4):1123–1125. 10.1097/SCS.0b013e3182902b3d 23851753

[13] Multi-Ethnic Pediatric Eye Disease Study G. Prevalence of myopia and hyperopia in 6- to 72-month-old african american and Hispanic children: the multi-ethnic pediatric eye disease study. Ophthalmology. 2010;117(1):140–147 e143. 10.1016/j.ophtha.2009.06.009 19926137 PMC2815146

[14] Robaei D, Kifley A, Gole GA, Mitchell P. The impact of modest prematurity on visual function at age 6 years: findings from a population-based study. Arch Ophthalmol. 2006;124(6):871–877. 10.1001/archopht.124.6.871 16769841

[15] Bothe N, Lieb B, Schafer WD. Development of impaired vision in mentally handicapped children. Klin Monbl Augenheilkd. 1991;198(6):509–514. 10.1055/s-2008-1046023 1895719

[16] Tay T, Martin F, Rowe N, Johnson K, Poole M, Tan K, et al Prevalence and causes of visual impairment in craniosynostotic syndromes. Clin Exp Ophthalmol. 2006;34(5):434–440. 10.1111/j.1442-9071.2006.01242.x 16872339

[17] Beaconsfield M, Walker JW, Collin JR. Visual development in the blepharophimosis syndrome. Br J Ophthalmol. 1991;75(12):746–748. 10.1136/bjo.75.12.746 1768667 PMC1042558

[18] Donahue SP, Arthur B, Neely DE, Arnold RW, Silbert D, Ruben JB, et al Guidelines for automated preschool vision screening: a 10-year, evidence-based update. J AAPOS. 2013;17(1):4–8. 10.1016/j.jaapos.2012.09.012 23360915

[19] Ottar WL, Scott WE, Holgado SI. Photoscreening for amblyogenic factors. J Pediatr Ophthalmol Strabismus. 1995;32(5):289–295. 8531032 10.3928/0191-3913-19950901-06

[20] Dirani M, Chan YH, Gazzard G, Hornbeak DM, Leo SW, Selvaraj P, et al Prevalence of refractive error in Singaporean Chinese children: the strabismus, amblyopia, and refractive error in young Singaporean Children (STARS) study. Invest Ophthalmol Vis Sci. 2010;51(3):1348–1355. 10.1167/iovs.09-3587 19933197 PMC3979485

[21] Arnold RW. Amblyopia risk factor prevalence. J Pediatr Ophthalmol Strabismus. 2013;50(4):213–217. 10.3928/01913913-20130326-01 23556991

[22] Wang J, Ding G, Li Y, Hua N, Wei N, Qi X, et al Refractive Status and Amblyopia Risk Factors in Chinese Children with Autism Spectrum Disorder. J Autism Dev Disord. 2018;48(5):1530–1536. 10.1007/s10803-017-3387-7 29170942

[23] Donahue SP, Arnold RW, Ruben JB, Committee AVS. Preschool vision screening: what should we be detecting and how should we report it? Uniform guidelines for reporting results of preschool vision screening studies. J AAPOS. 2003;7(5):314–316. 10.1016/S1091853103001824 14566312

[24] Fozailoff A, Tarczy-Hornoch K, Cotter S, Wen G, Lin J, Borchert M, et al Prevalence of astigmatism in 6- to 72-month-old African American and Hispanic children: the Multi-ethnic Pediatric Eye Disease Study. Ophthalmology. 2011;118(2):284–293. 10.1016/j.ophtha.2010.06.038 20888047 PMC3017730

[25] Borchert M, Tarczy-Hornoch K, Cotter SA, Liu N, Azen SP, Varma R, et al Anisometropia in Hispanic and african american infants and young children the multi-ethnic pediatric eye disease study. Ophthalmology. 2010;117(1):148–153 e141. 10.1016/j.ophtha.2009.06.008 19818509 PMC2814917

[26] Donahue SP. Relationship between anisometropia, patient age, and the development of amblyopia. Am J Ophthalmol. 2006;142(1):132–140. 10.1016/j.ajo.2006.02.040 16815261

[27] Chalmers R, Griffiths P. Is congenital nasolacrimal duct obstruction a risk factor for the development of amblyopia? British Orthoptic Journal. 1996;(53):29–30.

[28] Ingram RM. Refraction as a basis for screening children for squint and amblyopia. Br J Ophthalmol. 1977;61(1):8–15. 10.1136/bjo.61.1.8 836780 PMC1042865

[29] Gwiazda J, Marsh-Tootle WL, Hyman L, Hussein M, Norton TT. Baseline refractive and ocular component measures of children enrolled in the correction of myopia evaluation trial (COMET). Invest Ophthalmol Vis Sci. 2002;43(2):314–321. 11818372

[30] Robaei D, Rose KA, Kifley A, Cosstick M, Ip JM, Mitchell P. Factors associated with childhood strabismus: findings from a population-based study. Ophthalmology. 2006;113(7):1146–1153. 10.1016/j.ophtha.2006.02.019 16675019

[31] Abrahamsson M, Fabian G, Sjostrand J. Refraction changes in children developing convergent or divergent strabismus. Br J Ophthalmol. 1992;76(12):723–727. 10.1136/bjo.76.12.723 1486073 PMC504391

[32] Matsuo T, Matsuo C. The prevalence of strabismus and amblyopia in Japanese elementary school children. Ophthalmic Epidemiol. 2005;12(1):31–36. 10.1080/09286580490907805 15848918

[33] Goh P-P, Abqariyah Y, Pokharel GP, Ellwein LB. Refractive error and visual impairment in school-age children in Gombak District, Malaysia. Ophthalmology. 2005;112(4):678–685. 10.1016/j.ophtha.2004.10.048 15808262

[34] He M, Zeng J, Liu Y, Xu J, Pokharel GP, Ellwein LB. Refractive error and visual impairment in urban children in southern China. Invest Ophthalmol Vis Sci. 2004;45(3):793–799. 14985292 10.1167/iovs.03-1051

